# Immune checkpoint inhibitors in Wilms' tumor and Neuroblastoma: What now?

**DOI:** 10.1002/cnr2.1397

**Published:** 2021-05-01

**Authors:** Anders Valind, David Gisselsson

**Affiliations:** ^1^ Division of Clinical Genetics Lund University Lund Sweden; ^2^ Department of Pediatrics Skåne University Hospital Lund Sweden; ^3^ Department of Pathology Laboratory Medicine Skåne Lund Sweden

**Keywords:** immune checkpoint inhibition, neoantigens, neuroblastoma, Wilms' tumor

## Abstract

**Background:**

Therapeutic activation of tumor‐infiltrating lymphocytes using monoclonal antibodies targeting PD1 or PD‐L1 (immune checkpoint inhibitors—ICIs) has revolutionized treatment of specific solid tumors in adult cancer patients, and much hope has been placed on a similar effect in relapsed or refractory solid pediatric tumors. Recent clinical trials have disappointingly shown an almost nonexistent response rate, while case reports have demonstrated that some pediatric patients do achieve durable responses when treated with this type of drug.

**Aim:**

To elucidate this paradox, we mapped the landscape of expressed neoantigens as well as the levels of immune cell infiltration in the two most common extracranial solid pediatric tumors: Wilms tumor and neuroblastoma using state‐of‐the‐art in silico analysis of a large cohort of patients with these tumors.

**Methods:**

By integration of whole exome sequencing and RNA‐sequencing, we mapped the landscape of neoantigens in the TARGET cohorts for these diagnoses and correlated these findings with known genetic prognostic markers.

**Results:**

Our analysis shows that these tumors typically have much lower levels of expressed neoantigens than commonly seen in adult cancers, but we also identify subgroups with significantly higher levels of neoantigens. For neuroblastomas, the cases with higher levels of neoantigens were confined to the group without *MYCN*‐amplification and for Wilms tumor restricted to the *TP53‐*mutated cases. Furthermore, we demonstrate that neuroblastomas have an unexpectedly high level of CD8+ tumor‐infiltrating lymphocytes, even when compared to adult tumor types where ICI is an approved treatment.

**Conclusion:**

These results could be important to consider when designing future clinical trials of ICI treatment in pediatric patients with relapsed or refractory solid tumors.

**Abbreviations**
CD8: Cluster of differentiation 8
ICI: Immune checkpoint inhibition
MNA: MYCN amplified
MYCN: N‐myc proto‐oncogene
NBL: Neuroblastoma
ORR: Objective response rate
PD1: Programmed death 1
PD‐L1: Programmed death‐ligand 1
TCGA: The Cancer Genome Atlas
TIDE: Tumor immune dysfunction and exclusion
TP53: Tumor protein 53
WT: Wilms' tumor

## 1 INTRODUCTION

Immune checkpoint inhibitors (ICIs), targeting either programmed death‐1 (PD1) or programmed death‐ligand‐1 (PD‐L1), have revolutionized treatment of some tumor types in adults, such as lung cancer and melanoma. This success has fueled an interest in using ICI in relapsed and/or refractory pediatric cancers.^1^ At least three clinical studies evaluating the efficacy of ICI in this setting have recently been published^2^, ^3^, ^4^; disappointingly showing that the objective response rate for PD1 inhibition (pembrolizumab and nivolumab) and PD‐L1 inhibition (atezolizumab) for pediatric cancer patients is low. For pembrolizumab, the objective response rate (ORR) according to RECIST v1.1 for solid tumors was 5.5%.^2^ For nivolumab and atezolizumab, there was no objective response in any of the patients with solid tumors.^3^, ^4^ At the same time, case reports have shown that there exist pediatric patients that have a durable response on ICI, at least in combination with other antineoplastic agents.^5^ This apparent paradox could be due to the fact that in general, tumors in pediatric patients are less immunogenic than in adults, but that there exist patients with more immunogenic tumors that do respond to ICI. Children with such tumors would be strong candidates for future clinical trials.

## 2 METHODS

### 2.1 Processing of whole exome sequencing data

GRCh38‐aligned .bam‐files were downloaded from NCI Genomic Data Commons,^6^ for the TARGET‐WT and TARGET‐NBL cohorts to a secure computational cluster within the Division of Clinical Genetics at Lund University. The TARGET cohorts have previously been described in detail, including the stringent criteria used for ensuring samples of high technical quality. For the neuroblastoma cohort, we retained only Stage 4 high‐risk cases for all downstream analyses, as Stage 4S represents a special clinical and biological entity. Variant calling of single nucleotide variants (SNVs) and small insertions and deletions (indels) was performed using Mutect2^7^ version 4.1.3.0, according to the GATK4 best practice guidelines, with a normal reference panel consisting of all normal samples from the specific cohort. Filtering of putative variant calls was performed using the FilterMutectCalls tool from GATK4, including the ReadOrientationBiasFilter, to remove potential Oxo‐G artefacts,^8^ that are known to be present in a subset of TARGET neuroblastoma cases.^9^ Purity and ploidy estimation as well as detection of allele‐specific copy number alterations were performed using Sequenza,^10^ with standard settings. All proposed Sequenza solutions (purity and ploidy) were manually inspected to ensure the solution did not imply biologically unreasonable scenarios such as large‐scale homozygous deletions, and if so, refitted using one of the alternative solutions proposed by Sequenza.

### 2.2 Processing of RNASeq data

Bam‐files containing all sequencing reads from these experiments (ie, even reads that failed to map to the reference) were downloaded from the GDC. Raw paired end fastq‐files were extracted from these bam‐files using bazam.^11^ The raw reads where then pseudoaligned and transcript‐level abundances were quantified using kallisto.^12^ Transcript‐level abundances were merged into gene‐level estimates using the Bioconductor package tximport. For mutant allele expression detection, all variants underlying putative neoantigens were genotyped in their corresponding RNASeq bam‐file using freebayes,^13^ and variants with more than three reads supporting the ALT allele were considered expressed.

### 2.3 Combined cohort

The dataset named TCGA_TARGET_pancan was downloaded from UCSC Xena (UCSC toil data hub, dataset version 21 January 2017). We removed samples that were annotated to be from metastatic locations and also removed all primary brain tumor‐, leukemic‐, or lymphoma‐samples (the exact samples used are listed in Table S5). We then utilized QuantiSeq for estimation of infiltrating CD8+ TIL.

### 2.4 Clonality analysis

As we were only focused on primary tumors and typically only had access to data from a single sample per primary tumor, we opted to perform a simplified dichotomization of variants into clonal and subclonal instead of trying to perform a full clonal deconvolution. Briefly, we calculated the product of cancer cell fraction (CCF) and mutation multiplicity (m) following^14^ as:

$$u=\mathrm{CCF}\;*\;m=\mathrm{VAF}\;*\;\frac{\text{purity}\;*\;\mathrm{CNt}+\mathrm{CNn}-\mathrm{CNn}\;*\;\text{purity}}{\text{purity}}$$

where VAF is the variant allele frequency, CNt and CNn are the local absolute copy number for the tumor and normal sample, respectively, and m is defined as:

$$m=.\left\{\begin{array}{c} \text{if}\;u<1.0:1.0\;\\ \text{else}:\text{round}\left(u\right) \end{array}\right.$$

which was then used to calculate the fraction of cancer cells with a specific mutation (CCF).

This was followed by parametric bootstrapping assuming a binomial distribution of read counts to calculate a 95% confidence interval. Mutations whose 95% CI for cancer cell fraction contained 1.0 were classified as clonal, all others as subclonal.

### 2.5 Estimation of immune cell infiltration

The gene‐level abundance estimates from tximport were used as input to QuantiSeq,^15^ which was invoked through the immunedeconv^16^ package. QuantiSeq outputs estimated absolute fractions of 10 different immune cell populations; these absolute fractions allow for inter‐ and intrasample comparisons.

### 2.6 HLA‐typing, neoantigen prediction, and filtering

For typing of MHC‐I alleles (HLA‐A, HLA‐B, and HLA‐C), we utilized the PolySolver^17^ package. Neoantigen prediction was performed using NeoPredPipe,^18^ which utilizes NetMHCpan 4.0.^19^ All predicted neoantigens where filtered according to the criteria employed in Rosenthal et al,^20^ keeping only putative neoantigens with predicted binding affinity <500 nM and a rank percentage score <2%.

### 2.7 Classification of TCGA tumors into FDA approved and non‐FDA approved

We classified the tumors by whether their histology (regardless of PD‐L1/PD1 expression level) was included in the FDA‐approved indications for each of the following immune checkpoint inhibitors: Pembrolizumab (PD1), nivolumab (PD1), durvalumab (PD‐L1), atezolizumab (PD‐L1), and avelumab (PD‐L1). The TCGA breast cancer cohort was further subclassified according to each sample's PAM50‐signature, where a Basal classification was used as a proxy for triple‐negative breast cancer. These samples were included in the FDA‐approved cohort; all other breast cancer samples were classified into the non‐FDA‐approved group.

## 3 RESULTS

**FIGURE 1:**
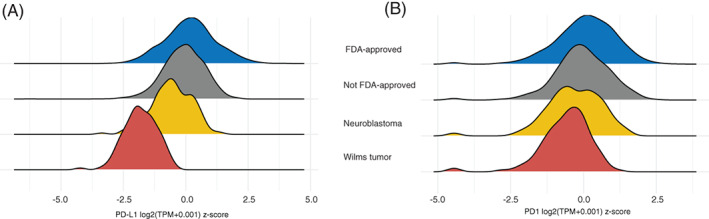
Comparative analysis of PD1/PD‐L1, CD8 TIL and expressed neoantigens in WT, NBL, and adult tumor cohorts. A, Ridge plot of the distribution of expression of PD‐L1 in WT, NBL, adult tumors with FDA approval for ICI, and adult tumors without FDA approval, showing the Z‐score of Log2‐transformed gene‐level transcript per million reads (TPM). Both WT and NBL have significantly lower levels of PD‐L1 than adult tumors (Table 1). B, The same comparisons as in A, for PD1 expression; see Table 1 for *P*‐values

**FIGURE 2:**
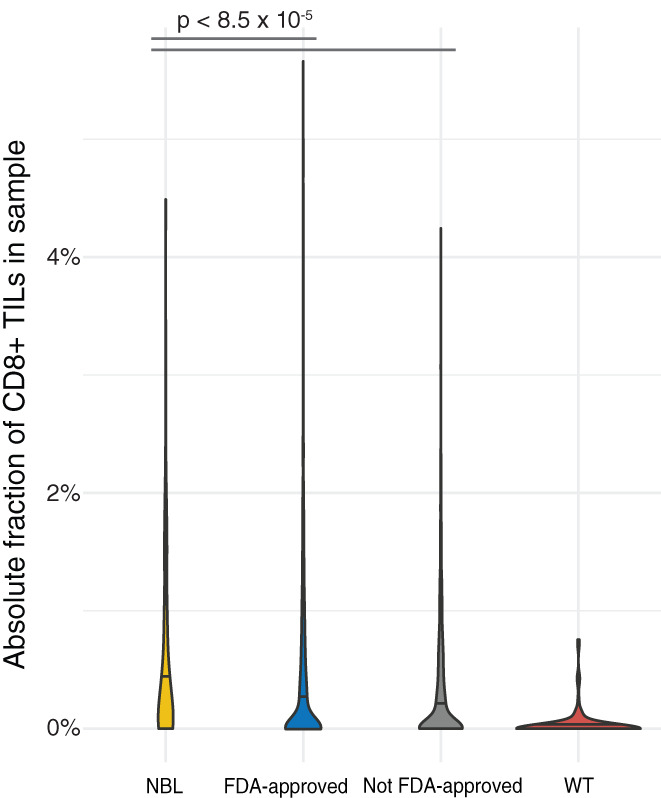
Violin plot of the absolute fraction of CD8+ TILs in the combined TARGET‐TCGA cohort (n = 2659 for the FDA‐approved group, and n = 1386 for non‐FDA approved group), showing that the absolute fraction of CD8+ TILs in NBL (n = 153) is highest among the four compared cancer groups and that WTs (n = 120) in general are devoid of CD8+ TIL; for a complete list of *P*‐values see Table 2

To assess the existence of such subgroups from a biological point of view, we set out to map the prevalence in solid pediatric tumors of biomarkers known from adult cancer to predict response to ICI. The most commonly used biomarkers for ICI response in adult cancers are tumor mutational burden (TMB), expressed neoantigens, PD‐L1 and PD1 protein expression, various gene signatures, microsatellite instability, and specific somatic genetic alterations.^21^ Large‐scale genomic analysis of pediatric solid tumors has consistently demonstrated very low TMBs compared to adult cancers.^22^ We thus chose to focus on evaluating PD1/PD‐L1 expression, the numbers of expressed neoantigens, as well as CD8+ TILs (tumor‐infiltrating lymphocytes) in the two most common extracranial solid tumors in children, Wilms' tumor (WT), and neuroblastoma (NBL). We started by analyzing the PD1/PD‐L1 mRNA expression across a combined cohort of patients from the WT and NBL TARGET (excluding Stage 4S NBL patients, as Stage 4S represents a distinct clinical entity with partly differing biology) cohorts and adult cancers from the TCGA pan cancer cohort, processed through a unified computational pipeline to ensure comparability.^23^ After keeping only primary solid extracranial tumors, this cohort consisted of 6083 patients. We grouped the TCGA tumors into cases from cancer types where there is an FDA approval for ICI and those where no such approval exists. This analysis revealed that both PD1 and PD‐L1 mRNA expression levels were significantly lower in the pediatric tumors than in adult cancers (Figure 1A,B). This was the case for adult cancers both with and without FDA approval of ICI (Mann‐Whitney *U* test; largest *P*‐value = 8,2 × 10^−4^, Table 1). Furthermore, cases with WT had significantly lower levels of PD‐L1 and PD1 than NBL. In concordance with this, immune cell deconvolution applied to the RNASeq data from the same set of samples revealed that the median level of CD8 TIL in WT was significantly lower than in NBL (Table 2). Surprisingly, the NBL cohort in fact had significantly higher levels of CD8+ TILs than both groups of adult tumors (Figure 2); the median absolute fraction of CD8+ TILs was more than three times higher than in the cohort of adult cancers with ICI approved by FDA, and more than four times higher than the median absolute fraction in the non‐ICI approved group.

**TABLE 1:** Comparison of mRNA levels for PD‐L1 and PD1 in TCGA to WT and NBL cases from TARGET, for a graphical representation of these data see Figure 1

Comparison	PD‐L1 *P*‐value	PD1 *P*‐value
FDA‐approved vs NBL	<2.2e‐16	5.898e‐07
FDA‐approved vs WT	<2.2e‐16	3.049e‐15
FDA‐approved vs non‐FDA‐approved	<2.2e‐16	4.554e‐08
Non‐FDA‐approved vs NBL	1.532e‐12	.00082
Non‐FDA‐approved vs WT	<2.2e‐16	4.144e‐11
WT vs NBL	<2.2e‐16	.007836

*Note*: All *P*‐values are from Mann‐Whitney *U* tests between the two groups designated in the comparison column.

**TABLE 2:** Estimated levels of CD8 infiltration in cases from the TCGA, WT, and NBL cases from TARGET, relates to Figure 2, all *P*‐values are from Mann‐Whitney *U* tests

Comparison	Group medians		*P*‐value
FDA approved vs NBL	0.00087	0.0027	.0008501
FDA approved vs WT	0.00087	0	<2.2e‐16
FDA approved vs non‐FDA approved	0.00087	0.00063	.003156
Non‐FDA approved vs NBL	0.00063	0.0027	5.593e‐06
Non‐FDA approved vs WT	0.00063	0	<2.2e‐16
WT vs NBL	0	0.0027	<2.2e‐16

**FIGURE 3:**
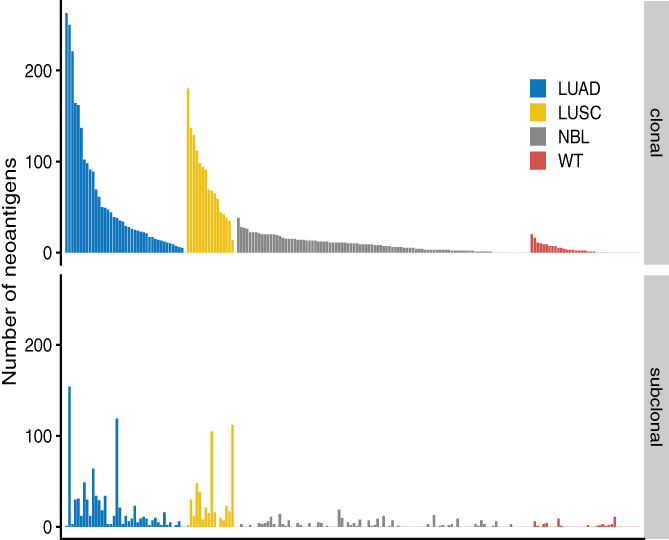
The number of expressed neoantigens based on clonality in the NBL (n = 99) and WT (n = 37) cohorts, compared to levels in lung adenocarcinomas (LUAD) and squamous cell carcinomas (LUSC) as presented in Rosenthal et al^20^

Next, we mapped the landscape of expressed neoantigens in NBL and WT. We annotated all expressed neoantigens as either clonal or subclonal based on allele‐specific copy number data, as clonal neoantigens are known to typically elicit a stronger immune response in lung cancer.^24^ For this analysis, we selected cases that had (a) whole exome sequencing data, (b) RNA sequencing generated on a uniform platform (Illumina), and (c) full clinical annotation, leaving a total of 37 cases with WT and 99 cases with NBL. We focused on samples taken prior to any systemic therapy to establish a baseline of neoantigen burden with minimal confounding by treatment by potentially mutagenic or immunomodulatory chemotherapy. Using well‐validated tools and established cut‐offs (see the methods section for details), we found a median of 3 (range 0‐22) expressed neoantigens in WT (summarized in Tables S2 and S4). There was a significantly higher number of clonal than subclonal neoantigens in WT (Wilcoxon signed rank test, *P*‐value = .012); the median number of clonal and subclonal neoantigens in WT was 2 (range 0‐20) and 0 (range 0‐11), respectively. In the NBL cohort, we found a median of 10 expressed neoantigens per sample (range 0‐38), which was significantly higher than in WT (Mann‐Whitney *U* test, *P*‐value = 8.64 × 10^−5^) and driven by differences in clonal (*P*‐value = 4.88 × 10^−5^) but not subclonal (*P*‐value = .178) expressed neoantigens. Similar to WT, there was a significantly higher number of clonal than subclonal neoantigens (Wilcoxon signed rank test, *P*‐value = 2.82 × 10^−11^); the median number of clonal neoantigens and subclonal neoantigens in NBL was 7 (range 0‐38) and 0 (range 0‐19), respectively. Comparing to adult cancers, these levels are significantly lower than the numbers seen in both lung adenocarcinoma and lung squamous cell carcinoma,^20^ as illustrated in Figure 3.

**FIGURE 4:**
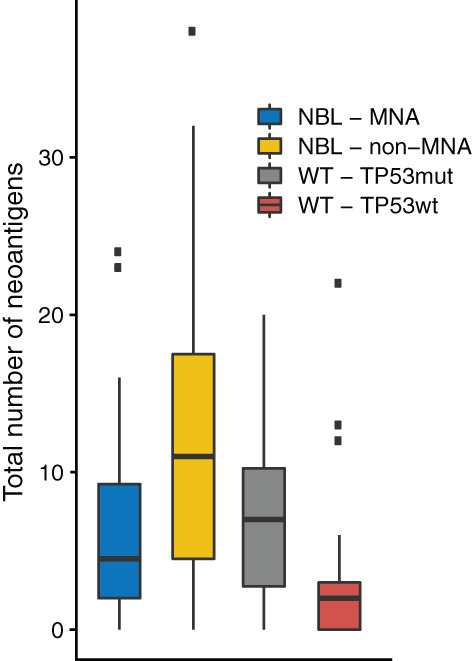
The distribution of neoantigens in the subgroup analysis of WT and NBL cases, showing higher numbers of expressed neoantigens in *TP53* mutated WT and non‐MNA NBL; for *P*‐values see main article text

We then proceeded by correlating the estimated levels of CD8+ TILs with the number of expressed neoantigens. For WT, we subdivided the cohort by *TP53* mutational status, as mutations in *TP53* are tightly linked to the prognostically unfavorable diffuse anaplastic subgroup of WT.^25^, ^26^, ^27^ Diffuse anaplasia is also associated to extensive intratumor heterogeneity and ongoing clonal evolution.^28^, ^29^ There was a significant correlation (Spearmans' rho = 0.52, *P*‐value = .041) between levels of CD8+ T‐cells and the total number of expressed neoantigens in *TP53*‐mutated cases. For *TP53*‐wild‐type WTs, no such correlation was found (Spearmans' rho = 0.064, *P*‐value = .780). *TP53*‐mutated cases had significantly higher numbers of neoantigens compared to *TP53*‐wild‐type cases (Mann‐Whitney *U* test *P*‐value = .014; Figure 4). There was no difference between PD1 and PD‐L1 mRNA levels in *TP53* mutated vs wild‐type cases.

In NBL, earlier studies have shown that cases with *MYCN* amplification (MNA), typically have lower levels of CD8+ TILs compared to cases without MNA.^30^ This was corroborated in our analysis, with a median absolute fraction of all cells in the sample of 1.1% in non‐MNA cases, and 0.6% in MNA cases (Mann‐Whitney *U* test, *P*‐value = .016). Interestingly, this difference was also seen in the number of neoantigens, in that tumors with MNA had significantly lower levels of total expressed neoantigens than tumors without MNA (median 4.5 vs median 11, Mann‐Whitney *U* test, *P*‐value = .0160; Figure 4 and Tables S1 and S3). This was driven by differences in the number of clonal neoantigens (median 3 vs 9, Mann‐Whitney *U* test, *P*‐value = .016) but not by any differences in the number of subclonal neoantigens (*P*‐value = .379). Furthermore, we discovered a moderate, but significant correlation between PD1 mRNA levels and clonal neoantigens in NBL cases without MNA (Spearmans' Rho = 0.23, *P*‐value = .048). The expression of PD1 was also elevated in non‐MNA cases vs MNA cases (*P*‐value = .011, Mann‐Whitney *U* test). No significant correlation between levels of PD‐L1 and clonal neoantigens was seen in the same group (Spearmans' Rho = −0.044, *P*‐value = .708). Finally, we used TIDE^31^ to generate T‐cell dysfunction and exclusion scores for each sample, in order to assess global T‐cell function for both the NBL and WT cohorts. Interestingly, for NBL, cases with MNA had significantly lower dysfunction scores (median − 0.778 vs 0.383, Mann‐Whitney *U* test, *P*‐value = 1.09e‐07), and higher exclusion scores (median 0.522 vs −0.221, Mann‐Whitney *U* test, *P*‐value 1.45e‐08), compared to non‐MNA cases. In the WT cohort, *TP53*‐mutated cases had significantly lower T‐cell dysfunction scores (−1.617 vs −1.501, Mann‐Whitney *U* test, *P*‐value = .045) but did not differ in exclusion scores (Mann‐Whitney *U* test, *P*‐value = .15).

## 4 DISCUSSION

To summarize, we found that in cohorts of high‐risk primary tumors of the two most common extracranial solid malignancies in children, there exist subgroups with higher neoantigen burden (*TP53*‐mutated WT and non‐*MYCN*‐amplified NBL). However, even among these, the levels of expressed neoantigens are far from those observed in adult tumor types where ICI is an approved treatment modality. Furthermore, the biological interplay between immune response and *TP53* mutations is tumor‐type dependent and complex,^32^ and further work is clearly needed to unravel the how *TP53*‐mutated WT interplay with the host immune system. The level of PD‐L1‐expression in WT and NBL was also significantly lower than in adult tumors where ICI is commonly used. We found, rather unexpectedly, that CD8+ TIL levels in NBL are on the same level as in some adult tumors, but that PD1 and PD‐L1 expression are still significantly lower. This could potentially be due to low levels of MHC‐1 expression seen in NBL,^33^ prohibiting the chronic stimulation of CD8+ TILs that causes them to up‐regulate PD1.^34^ However, the correlation between clonal neoantigens and PD1 expression levels in non‐MNA NBL cases argues against this model and could signal that there in fact exists an immune response of activated CD8+ TILs targeting these neoantigens. Whether this response is of sufficient magnitude to be modulated with significant clinical effects remains to be seen. One interesting ongoing study is the INFORM2‐NivEnt,^35^ which combines ICI treatment with an HDAC‐inhibitor in relapsed pediatric cancer patients, in four biomarker defined groups, one of which is based on PD‐L1 expression. This could in theory help to delineate subgroups who do respond to ICI. Another interesting avenue is using ICI as an adjunct to the anti‐GD2 monoclonal antibodies that are used in current treatment of high‐risk NBL, where a recent case report shows intriguing data.^5^ Our TIDE analysis indicates that in NBL, MNA cases might have evolved immune escape mechanisms, potentially mediated through down‐regulation of MHC‐1 expression, which is a known feature of MNA NBL.^30^ For non‐MNA cases, the higher dysfunction scores estimated by TIDE could signify that the increased levels of neoantigens seen in this group is still insufficient to generate a functional immune response.

While recognizing that the gold standard for clinical efficacy is properly conducted randomized controlled trials, we argue that thorough consideration of the data we present here is important when designing such trials aiming at evaluating ICI in WT and NBL. Our data indicate that NBL and WT patients with a potential benefit from ICI are most likely confined to small subgroups of patients, nested within the groups of *TP53* mutated WTs and non‐MNA high‐risk NBLs, respectively. Because the main candidates for ICI in pediatric oncology will be patients where conventional first‐line therapy has failed, our analyses also point to the need for more extensive molecular immuno‐oncological data from relapsing and treatment refractory tumors. Such datasets could, in addition to delineating additional patient subgroups likely to benefit from ICI, also be useful in a comparative analysis with the data presented in this paper to probe how standard treatment regimens modulate the levels of CD8+ TILs and expressed neoantigens.

## CONFLICT OF INTEREST

The authors report no conflict of interest.

## AUTHOR CONTRIBUTIONS

*Conceptualization; data curation; formal analysis; investigation; methodology; project administration; resources; software; visualization; writing‐original draft; writing‐review & editing*, A.V.; *Conceptualization; funding acquisition; supervision*, D.G.

## ETHICAL STATEMENT

The study was approved by the regional ethics review board under permit numbers L289‐11 (genomic analyses; updated as L796‐2017).

## Supporting information


**Table S1.** Neuroblastoma expressed mutations with clonality info and neoantigen classification.

**Table S2.** Wilms Tumor expressed mutations with clonality info and neoantigen classification.

**Table S3.** Clinical info, genomic subgroups, CD8+ infiltration, purity and TIDE results for the Neuroblastoma Cohort

**Table S4.** Clinical info, genomic subgroups, CD8+ infiltration, purity and TIDE results for the Wilms Tumor Cohort.

**Table S5.** TCGA Samples used for comparison and their classification according to FDA approved ICI treatment.

## Data Availability

Raw data can be obtained through the controlled access mechanism at dbGAP (accession phs000218). Processed data (neoantigen counts, clinical info, CD8+ TIL‐levels) are available in the supplementary tables to this article.
